# Coherent amplification of X-ray scattering from meso-structures

**DOI:** 10.1107/S2052252517008107

**Published:** 2017-07-10

**Authors:** Julien R. Lhermitte, Aaron Stein, Cheng Tian, Yugang Zhang, Lutz Wiegart, Andrei Fluerasu, Oleg Gang, Kevin G. Yager

**Affiliations:** a Center for Functional Nanomaterials, Brookhaven National Laboratory, Upton, New York, NY 11973, USA; b National Synchrotron Light Source II, Brookhaven National Laboratory, Upton, New York, NY 11973, USA; c Department of Chemical Engineering, Columbia University, New York, NY 10027, USA; d Department of Applied Physics and Applied Mathematics, Columbia University, New York, NY 10027, USA

**Keywords:** coherent amplification, small-angle X-ray scattering, meso-structures

## Abstract

Small-angle X-ray scattering (SAXS) often includes an unwanted background, which increases the required measurement time to resolve the sample structure. This is undesirable in all experiments, and may make measurement of dynamic or radiation-sensitive samples impossible. Here, we demonstrate a new technique, applicable when the scattering signal is background-dominated, which reduces the requisite exposure time. Our method consists of exploiting coherent interference between a sample with a designed strongly scattering ‘amplifier’. A modified angular correlation function is used to extract the symmetry of the interference term; that is, the scattering arising from the interference between the amplifier and the sample. This enables reconstruction of the sample’s symmetry, despite the sample scattering itself being well below the intensity of background scattering. Thus, coherent amplification is used to generate a strong scattering term (well above background), from which sample scattering is inferred. We validate this method using lithographically defined test samples.

## Abstract 2

A new technique is presented to allow the measurement of weakly scattering samples despite a high-background environment. Coherent interference between the sample and a nanofabricated ‘amplifier’ structure generates a strong interference scattering pattern, which can be analyzed using angular correlation functions to reconstruct the sample’s symmetry.

## 1. Introduction   

Designed nanomaterials hold the promise of vastly improved functional response for a range of demanding applications. The key to controlling functionality in these materials lies in the precise control over the spatial arrangement of nano-objects (*e.g.* nanoparticles), whose collective properties give rise to tailored optical (Fan *et al.*, 2010 ▸; Jones *et al.*, 2011 ▸), electrical (Tapio *et al.*, 2016 ▸), or catalytic response (Daniel & Astruc, 2004 ▸). A variety of methods have been developed to precisely control the positioning of nanoscale components. Lithography using light, electrons, or ions, can be used to precisely scribe desired structures. Self-assembly can be used to direct the organization of nanoparticles, forming complex motifs ranging from extended three-dimensional superlattices (Mirkin *et al.*, 1996 ▸; Murray *et al.*, 2000 ▸; Redl *et al.*, 2003 ▸; Park *et al.*, 2008 ▸; Nykypanchuk *et al.*, 2008 ▸; Zhang *et al.*, 2013 ▸; Ye *et al.*, 2015 ▸), to finite-size clusters (Sharma *et al.*, 2009 ▸; Lo *et al.*, 2010 ▸; Vial *et al.*, 2013 ▸; Tan *et al.*, 2014 ▸; Zhang *et al.*, 2014 ▸; Tian *et al.*, 2015 ▸). A key challenge to continued progress in nanomaterials design is the structural characterization of these mesoscale objects. Characterization is especially problematic for self-assembled systems exploiting soft linkers and a solvent (often aqueous) environment, since removal from this environment typically disrupts the ordering. Measurement in the native environment is thus highly desirable, but this limits one to measurement probes with long penetration depths and small wavelengths. X-rays are particularly well suited for the task, meeting both these criteria. Small-angle X-ray scattering (SAXS) is routinely used for the measurement of the structure of extended superlattices, for example Jones *et al.* (2010 ▸), Yager *et al.* (2014 ▸), Tian *et al.* (2015 ▸) and Senesi & Lee (2015 ▸).

The *in-situ* measurement of finite-sized meso-objects, which are inherently small and weakly scattering, remains a serious challenge. The sample environment (*i.e.* solution) introduces significant background scattering. This greatly reduces the overall signal-to-noise ratio per measurement, increasing the required measurement time. This is especially problematic for radiation-sensitive materials, such as DNA-mediated nanoparticle superlattices, which degrade under long X-ray exposure. Here, we propose a new technique to overcome the limitation of background-dominated measurement: coherent X-ray amplification of sample scattering above background (X-amp).

Our method consists of boosting the effective sample signal by ensuring coherent interference between it and a designed strongly scattering object. Our work builds upon previous work in coherent interference, signal boosting (Shintake, 2008 ▸), and related work in Fourier transform holography (McNulty *et al.*, 1992 ▸; Eisebitt *et al.*, 2004 ▸). Importantly, as subsequently shown by Schropp & Schroer (2010 ▸), coherent interference cannot boost the intrinsic signal-to-noise ratio of a particular scattering entity. However, interference can boost a weak signal to be above an otherwise dominating background. Although the sample scattering itself may be well below the background, the ‘amplifier’ structure can be designed to scatter very strongly; the coherent interference between the sample and amplifier can thus be designed to be above background, even though the sample scattering alone is not. The interference signal appears as fringing of the total scattering pattern, in the *q*-ranges where both the sample and amplifier scatter. While the spacing of the fringes themselves merely encodes the spatial separation between the sample and amplifier (which is generally not of interest), the distribution of fringes throughout *q*-space allows the scattering pattern of the sample to be inferred. Thus, the interference scattering encodes information about the sample structure. This information can be robustly extracted by applying correlation methods, which are well suited to identifying sample symmetry.

Measuring the symmetry of X-ray scattering rings reveals additional information about structural order beyond that available in the isotropic scattering pattern. For extended periodic materials (*e.g.* superlattices), the multiplicity of each scattering peak is a unique signature of the lattice type. Per-peak symmetry information can thus resolve ambiguities between otherwise similar scattering patterns. Moreover, lattice identification is typically impossible when only a single scattering peak is measurable (due to low signal, sample disorder, *etc*.); whereas the inclusion of symmetry information can distinguish different candidate structures. For example, the symmetries of the first-order peak for f.c.c. and h.c.p. lattices are distinct (4-fold *versus* 6-fold). More broadly, symmetry analysis probes crucial information about local packing motifs (Wochner *et al.*, 2009 ▸; Altarelli *et al.*, 2010 ▸) and orientational order in nanomaterials (Lehmkühler *et al.*, 2014 ▸, 2016 ▸), which is especially important since the collective properties exhibited by such materials depend strongly on the local ordering (nearest- and next-nearest-neighbor interactions). Finite-size meso-clusters represent another important class of nanomaterials. Precise arrangements of nanoparticles can now be generated using DNA origami frames (Liu *et al.*, 2016 ▸). Because of the finite size, these materials do not exhibit sharp structural peaks, but instead exhibit complicated form factor oscillations. By resolving the symmetry of the scattering features, one can uncover the underlying structural motifs of the assembly, and thus reconstruct the meso-cluster structure. Thus, the measurement of symmetry provides crucial information for periodic, disordered and finite-size nanomaterials. There is a pressing need to be able to measure nanoscale order in regimes of extremely low signal to noise: for organic materials that scatter weakly, in solvent environments with high background, and in kinetic series where each individual frame may have very few photon counts. It is within this context that improvements in the signal-to-noise ratio of angular correlation methods have enormous value.

In this work, we demonstrate how a modified angular correlation technique can be used to extract sample symmetry from the coherent interference term. This enables measurement of weakly scattering samples, and moreover allows measuring sample symmetry (and thus local packing motifs) beyond the ensemble limit. That is, one can measure aspects of sample structure that are normally averaged-out in bulk scattering from a large, isotropic distribution of meso-objects. By boosting the effective signal-to-noise ratio of weakly scattering samples, X-amp should make feasible a host of previously impossible experiments, including the measurement of individual meso-clusters in high-background solution environments, the reconstruction of three-dimensional scattering patterns of finite-sized entities, and *in-situ* measurement of the dynamics and kinetics of self-assembling nanomaterials.

## 2. Methods   

### 2.1. Experimental setup   

**Figure 1:**
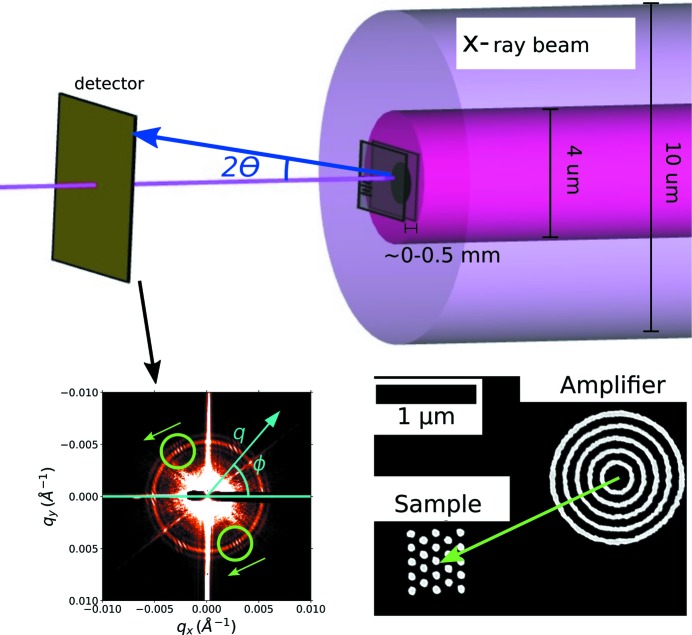
Experimental setup. The amplifier (ring structure) is placed upstream of the sample of interest. The X-ray beam is denoted by the large purple cylinder; the inner cylinder denotes the approximate transverse coherence length (the longitudinal coherence length is large). When the sample and amplifier are within the same coherence volume, the scattering pattern (lower left) exhibits fringes (circled in green) whose spacing and direction are related to the transverse separation between the two scattering entities (green arrow). The lower-right figure shows an SEM image of a validation structure, where the sample and amplifier were patterned on a single substrate.

Synchrotron experiments were performed at the coherent hard X-ray (CHX, 11-ID) undulator beamline at the National Synchrotron Light Source II (NSLS-II) at Brookhaven National Laboratory (Fluerasu *et al.*, 2011 ▸; Chubar *et al.*, 2013 ▸; Lhermitte *et al.*, 2017 ▸). The X-ray energy was set to 8.9 keV (1.4 Å wavelength) using a double-crystal monochromator (energy resolution 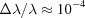). The partially coherent beam was focused 0.5 m upstream of the sample position to a spot size of 10 µm using a set of compound refractive lenses (CRL) and kinoform lens (KL) closer to the sample (Chubar *et al.*, 2013 ▸). The sample–detector distance was set to 4.81 m. The diffractometer used for sample mounting features two independent stacks of stages (each with three translational degrees of freedom), allowing for independent alignment and motion of the sample substrate and amplifier substrate. For the proof-of-principle measurements with the sample and amplifier fabricated on a single substrate, only a single motion stack was required. The experimental setup is shown in Fig. 1 ▸.

High beam coherence was ensured by measuring a sample of known scattering and ensuring the visibility of the expected fringes for the same experimental setup. In this case, the sample was a two-dimensional array of dots approximately 2.6 µm in extent (see supporting information), while beam transverse coherence length is estimated to be 4 µm.

### 2.2. Sample preparation   

The sample and amplifier structures were fabricated using electron-beam lithography (Jeol-JBX6300-FS). In both cases, gold nanostructures were fabricated on 150 µm thick silicon substrate [using a similar protocol as in our previous work (Lhermitte *et al.*, 2017 ▸)]. The samples consist of a hexagonal arrangement of dots, while the amplifier consists of concentric rings. The radial periodicity of the rings scatters photons into certain regions of *q*-space, increasing the scattered signal in these regions. Circularly symmetric rings – as opposed to two-dimensional arrays – were chosen to produce azimuthally (ϕ) isotropic scattering. The ring spacing is designed such that the resultant scattering peak closely overlaps the scattering peaks for the sample of interest (refer to the supporting information for additional details). Samples were imaged using scanning electron microscopy (SEM) to confirm their structure and quality. Fig. 1 ▸ shows a validation structure where the sample and amplifier are patterned on the same substrate. Similar quality of structures were obtained when the sample and amplifier were patterned on separate substrates.

## 3. Theory   

The X-ray scattering signal measured on a detector follows shot–noise counting statistics (Goodman, 1985 ▸). For a measured intensity *I* for some pixel and exposure time 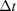, with a mean counting value of 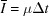 (where μ represents the counts for some pixel per exposure time), the measured signal is simply 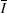. The noise is the variance 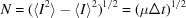 where the last step assumes that the intensity follows Poisson statistics and the angle brackets denote an ensemble average over measurements (Goodman, 1985 ▸). The signal-to-noise (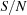) ratio is: 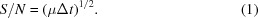
For a fixed solid angle, the quality of a diffraction pattern strongly depends on the exposure time. Typically, a good value of signal-to-noise ratio for a collection of pixels is on the order of 1, so that the average number of photons measured in a given pixel is near 1. Higher criteria exist, such as a signal-to-noise ratio of 4 or 5, which results in 16 to 25 photons per pixel (Rose, 1974 ▸). For a desired signal-to-noise ratio of 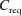, the exposure time required scales as 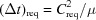.

When a background is introduced, the mean intensity becomes 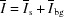 where the subscripts s/bg refer to the sample/background. The signal must now be discernible from the background, and so the signal is the difference between the sample and a measured background 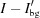, where 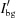 is a measured background. In the ideal case where the background is perfectly measurable 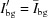, the signal is the average of this quantity: 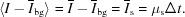
The noise is the variance of this quantity: 
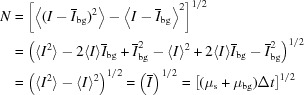
The signal-to-noise ratio is then: 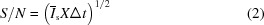


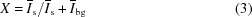


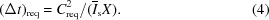
Thus, to achieve the same signal-to-noise ratio of a diffraction pattern with the presence of a background, one would need to expose 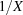 times longer than one would have needed to successfully measure the sample on its own. In the limit of high backgrounds, this reduces to 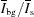. The increase in measurement time is of course inconvenient. In the case of radiation-sensitive or time-varying samples, the large requisite exposure time may in fact make the measurement impossible.

### 3.1. Boosting by coherent interference   

The intensity of coherently scattered photons, in the far field, is given by the Born approximation (Als-Nielsen & McMorrow, 2011 ▸), and proportional to the modulus squared of the form factor: 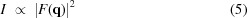


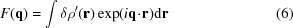
where 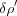 is an effective electron density difference taking into account the medium electron density as well as energy-dependent dispersion corrections (see the supporting information for additional details). If we introduce an amplifier within the coherence volume of a sample, equation (6) still holds, and the scattered field may be broken into the integrals centered over the sample and the amplifier volume at positions 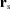 and 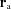, respectively: 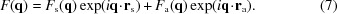
The resultant intensity is then: 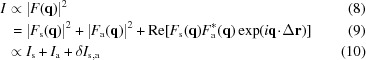


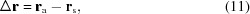
where 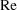 denotes the ‘real part of’. The coherent interference results in an interference term 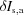 which encodes a combination of sample 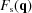 and amplifier 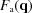 form factors. This interference term contains sets of fringes determined by the relative distance 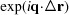 of sample and amplifier.

A simple example of this effect can be seen by examining the scattering in Fig. 1 ▸ (lower left) of a sample and amplifier (SEM in lower right). In addition to the sum of the diffraction patterns from the sample and amplifier seen, one observes fringes where the scattering patterns of the two overlap [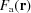 and 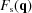 nonzero].

The direction of the fringes is along the center to center distance of the sample to amplifier (where the center is defined by the form factor) with fringe spacing inversely proportional to the sample-to-amplifier distance.

If background scattering is present, the overall scattering intensity will include this contribution. Assuming that there is no discernible coherent interference between the sample and background, the contribution is purely additive: 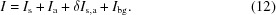
Examples of backgrounds like this are the scattering of windows outside the coherence volume, or solvent molecules within the coherence volume fluctuating on time scales much faster than the measurement time. When the scattering (of sample, amplifier, and background) is static, the noise of such a term is simply due to Poisson statistics: 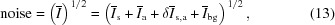
where an overbar indicates an average over time. To gain an intuitive sense of the signal, we make the assumption that the sample and amplifier are centrosymmetric, so their form factors are real. The interference term is then: 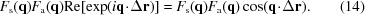
The sample and the amplifier will generally be spatially separated by a length greater than their overall sizes; thus the phase term will oscillate more rapidly than the variations in reciprocal space of the sample or amplifier term. Thus the ‘signal’ from the interference term (in terms of 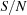) will be the difference between the maximum and minimum of the fringes observed, or: 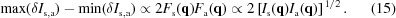
The signal-to-noise ratio is then: 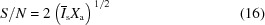


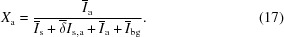
In the case of a weak sample (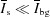) and strong amplifier (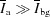), the resultant signal-to-noise ratio approaches an asymptotic value of 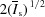. Contrasting this to the signal-to-noise ratio for a sample without amplifier, which approaches 0 in the limit of large background, this is quite a gain. On the other hand, this signal-to-noise ratio may not improve upon further increasing the amplifier signal beyond that of twice the signal-to-noise ratio of the sample in the absence of background [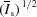]. Two important conclusions may be drawn from this. First, in regimes of strong background, a strong amplifier may be used to improve the signal-to-noise ratio of a sample. Second, the amplifier cannot amplify the sample’s signal much beyond what it would have been had the sample been measured on its own without a background [equation (2)]. This has also been more rigorously shown by Schropp & Schroer (2010 ▸). Thus, the utility of the proposed X-amp method is not to amplify the sample scattering per se, but rather to boost its signal above a background that would otherwise obscure it.

### 3.2. Angular correlations   

Angular correlation analysis can be used to extract symmetry from noisy X-ray scattering data (Kurta *et al.*, 2012 ▸; Altarelli *et al.*, 2010 ▸; Latychevskaia *et al.*, 2015 ▸). One typically measures multiple realizations of the sample over time and averages quantities that are preserved across the ensemble of samples (in this case angular symmetries). The angular correlation is defined as: 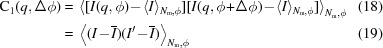
where, for notational simplicity 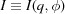, 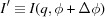, and 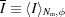 and 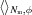 denotes an average over angle and measurements. In the event of large backgrounds, the signal-to-noise ratio scales unfavorably, as 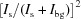 (Kirian *et al.*, 2011 ▸; Lhermitte *et al.*, 2017 ▸). In the limit of a strong amplifier (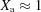), the sample signal-to-noise ratio no longer depends on background. One expects the same gains in the corresponding angular correlation functions. A sensible approach to attempt to improve this scaling is then to compute angular correlations on the amplified X-ray scattering pattern. However, it turns out that due to the extra phase 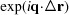 in the interference term, this correlation function averages out to zero when averaging over many sample realizations (see Appendix A for details). Rather, a higher-order correlation function is necessary: 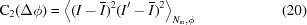


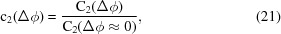
where 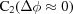 refers to taking the correlation function near zero [as explained in Lhermitte *et al.* (2017 ▸)]. Assuming that the amplifier is static and that the background dominates the sample scattering, so that 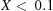, the correlation function is approximated to be: 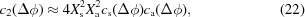
where 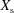 and 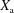 are the ratio of the sample and amplifier scattering to total scattering, respectively (proof is provided in appendix 8). Intuitively, as there is a visible improvement in the diffraction patterns by eye, one would also expect a signal-to-noise improvement in the sample’s angular correlations. This will be made explicit in later sections of this paper.

## 4. Results   

**Figure 2:**
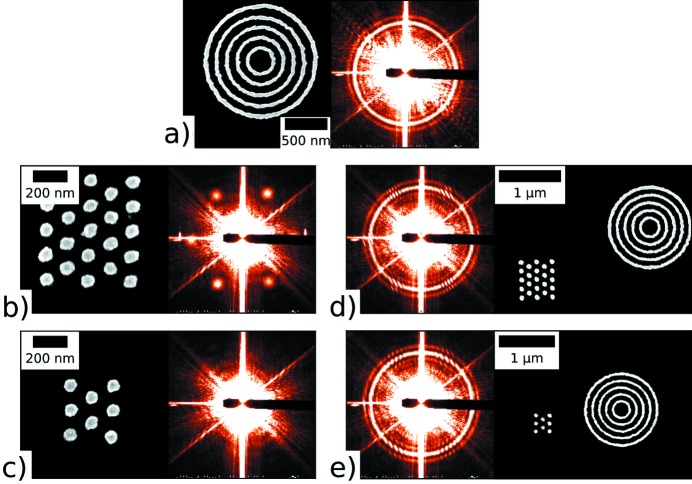
SEM images and SAXS data for a selection of meso-structures. (*a*) A pattern of concentric rings generates a strong isotropic ring of scattering; this structure is used as an amplifier. (*b*) A 5 × 5 hexagonal arrangement of 23 dots, fabricated as a test sample. (*c*) A 3 × 3 hexagonal arrangement of 8 dots, which acts as a very weakly scattering sample. (*d*) When there is coherent interference between a sample and the amplifier (in this example, by patterning them side by side on a single substrate), fringes appear in the scattering pattern, wherever both sample and amplifier scatter strongly. (*e*) In the case of a very weakly scattering sample (which cannot be discerned above the experimental background), coherent interference with the amplifier nevertheless generates a visible fringe pattern. In other words, although the sample alone cannot be resolved, coherent amplification can be used to infer the sample’s scattering pattern.

In order to provide a proof-of-principle validation of the X-amp technique, we first fabricated sample/amplifier pairs where both structures are patterned side by side on a single substrate (Fig. 1 ▸, lower right). This enables us to test coherent interference, and associated signal amplification, under idealized conditions; it guarantees that the sample and amplifier are co-aligned with respect to the beam, within the same coherence volume. Samples were located and aligned to the beam center (to within 500 nm) using strongly scattering fiducial structures patterned on the substrates (refer to the supporting information for details on fabrication layout, and procedures for locating and aligning structures). The test samples were arrays of dots arranged into a two-dimensional hexagonal lattice. Coherent interference was exploited by placing these test samples with amplifiers consisting of a set of concentric rings. The ring spacing was designed to match the spacing of planes of the sample arrays, such that their scattering peak coincides with the first-order peak from the sample. Many variants of the samples [5 × 5 (Fig. 2 ▸ *b*), 4 × 4 and 3 × 3 (Fig. 2 ▸ *c*) arrays] and amplifiers (5, 7 or 10 concentric rings) were measured; representative results are presented below. The hex arrays were patterned nearby the amplifier structures, with multiple copies of the sample/amplifier pair fabricated on a single substrate (randomly varying the sample orientation) to simulate an ensemble of samples.

Examples of meso-objects probed using X-ray scattering are shown in Fig. 2 ▸. As can be clearly seen, the effect of the interference between the sample and amplifier is to produce a fringed pattern in the parts of *q*-space where both the sample and amplifier scatter strongly. For instance, interference fringes can be seen in Fig. 2 ▸(*d*), where both the amplifier (ring of scattering, Fig. 2 ▸ *a*) and sample (six scattering peaks, Fig. 2 ▸ *b*) scatter. Fig. 2 ▸(*c*) shows the scattering pattern for an extremely small meso-object: an array of exactly eight nanoparticles. This ultra-small meso-object scatters so weakly that its scattering pattern is not discernible above the experimental background. However, in the presence of an amplifier, the sample’s scattering peaks become visible as fringed regions of the total scattering pattern (Fig. 2 ▸ *e*). This qualitatively confirms that X-amp can allow the measurement of samples whose signal-to-noise ratio is below the experimental background.

### 4.1. Angular correlations   

From the fringing observed in the scattering of a sample in the presence of an amplifier, one can qualitatively discern the structure of the sample scattering. However, to robustly reconstruct the sample scattering, we exploit angular correlation analysis. Angular correlations are, by construction, insensitive to absolute orientation. Thus, although replicate measurements of a sample at many different orientations averages out the orientational (symmetry) information, accumulation of the correlation functions instead reinforces the orientational symmetry. This allows one to reduce image exposures to a time shorter than the dynamics of the sample and average their angular correlations to extract meaningful information about their symmetry. Thus, in the X-amp method, instead of reconstructing the sample’s reciprocal space, we reconstruct the sample’s angular correlation map, which provides a robust descriptor of sample structure.

**Figure 3:**
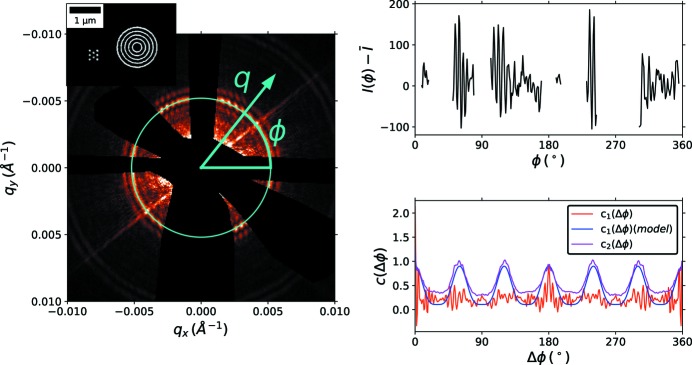
Diffraction pattern measured for a sample (3 × 3 hexagonal array) in the presence of an amplifier (concentric ring structure). The corresponding SEM is shown in the upper left. The ring along the blue contour is remapped to a (average-subtracted) one-dimensional curve *versus* ϕ (top right). The first- and second-order angular correlations are plotted in the lower-right figure. The first-order correlation (red) is flat and noisy. However, the second-order correlation function (magenta) closely matches the idealized expectation for the known sample symmetry (blue). The interference fringes oscillate as the inverse sample–amplifier distances ≃ 2π/1 µm ≃ 6 × 10^−4^ Å^−1^.

Fig. 3 ▸ shows an example of this angular correlation analysis, where the correlation functions for nine different orientations of a sample are accumulated. We use our previously described analysis pipeline (Lhermitte *et al.*, 2017 ▸), which accounts for image masking artifacts, but use the higher-order 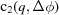 correlation function.

The first-order correlation function (*c*_1_, red) does not obviously reproduce the expected sample symmetry. The structure in the curve is encoding the correlated (signed) intensity variations of the interference term, including its random phase. Averaging over multiple realisations then averages out to a flat curve containing no information. The second-order correlation function (*c*_2_, magenta), however, robustly reconstructs the sample symmetry, as can be seen by its close match to the computed idealized correlation curve (blue). In the case of the second-order term, the correlation is capturing the absolute (strictly positive) intensity deviations of the interference term. Because the phase of the interference term is random (between different realisations), the average of the second-order correlation function reconstructs the *envelope* of the interference term. Thus, the second-order correlation function reproduces the underlying sample symmetry.

### 4.2. Overcoming background   

**Figure 4:**
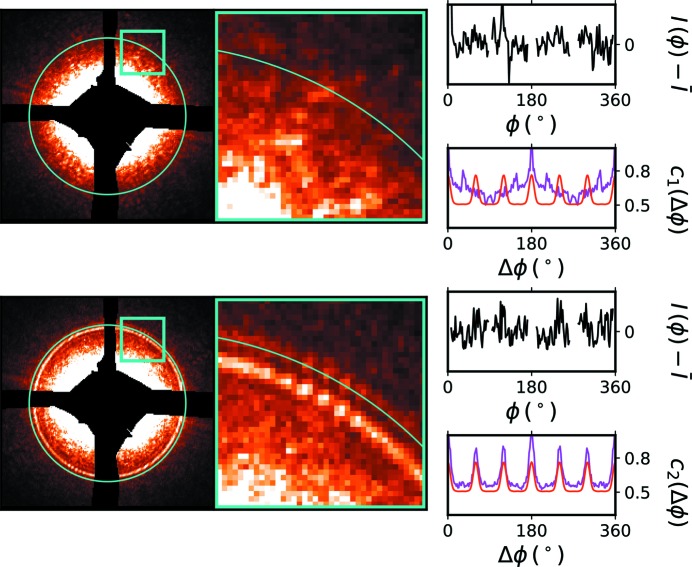
Example of a sample (5 × 5 hex array) where the background has been intentionally increased (using the diffuse scattering of 3 × 75 µm of polyimide material). The top row shows data for the sample alone. The sample’s scattering peaks (which should appear along the cyan circle) are not discernible (the blue square shows a zoomed-in region). The intensity along this azimuthal arc (black curve, right) similarly shows no hint of the sample peaks. Performing a correlation analysis (magenta curve) does not recover the sample symmetry (which should match the model curve, shown in red). The bottom row shows an equivalent sample in the presence of an amplifier. Although the sample peaks still cannot be seen, distinct interference fringes are clearly visible. The angular intensity (black curve) can be analyzed to extract the second-order correlation function (magenta), which closely matches the expected sample symmetry (red). Thus, the interference fringes encode the sample’s structural information. By exploiting interference between the sample and an amplifier, the sample’s structure can be reconstructed, even though the sample by itself cannot be measured.

To test the ability of coherent interference to combat background scattering, we performed a sequence of experiments where the background was intentionally increased by introducing sheets of a polyimide film known to generate diffuse scattering. These sheets were placed a few centimeters downstream of the sample, well outside the ∼500 µm requirement. Fig. 4 ▸ shows data for a weakly scattering sample, with substantial background scattering (3 × 75 µm of polyimide). With this background, the sample itself cannot be discerned visually; nor can the sample symmetry be recovered using a correlation analysis (Fig. 4 ▸, top). In short, the sample is unmeasurable. However, when an identical sample is in the presence of an amplifier, one can clearly distinguish both the amplifier scattering, and also the fringes resulting from the sample–amplifier interference. Performing a correlation analysis on this data (averaging over nine realizations with different sample orientations), we recover the correlation function and thus the hexagonal symmetry of the sample (Fig. 4 ▸, bottom). This experimentally validates the proposed method, allowing unmeasurable samples to be boosted above the background noise.

### 4.3. Signal-to-noise estimates from simulations   

**Figure 5:**
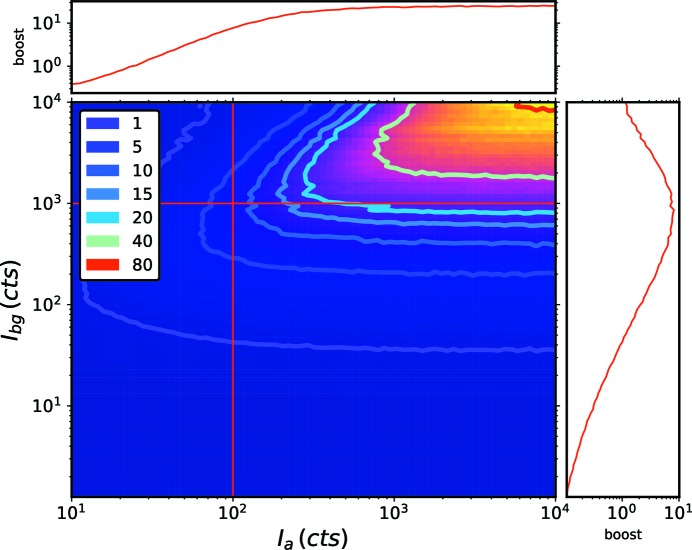
We define a boost factor as the ratio of the signal to noise obtained with coherent amplification to that obtained without using an amplifier. The boost factor thus quantifies the experimental gain that can be achieved by exploiting X-amp. The plot false color map shows the boost factor for simulations using constant scattering of the sample (10 counts s^−1^ in the data shown), but varying the scattering intensity of the background (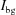) and amplifier (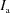).

The improvements in the signal-to-noise ratio of angular correlations using an amplifier have so far been qualitative. We provide here a brief demonstration of the signal-to-noise gains using the amplification scheme in the presence of an unstructured background. We use simulated scattering data in order to quantify the scaling over a wide parameter space; the simulation procedure is similar to that used in our previous report (Lhermitte *et al.*, 2017 ▸), and elaborated in the supporting information. The signal-to-noise ratio is computed for two cases: (1) the computation of the 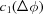 correlation of the sample on its own in the presence of a background; (2) the computation of the 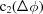 correlation function of the same sample in the presence of an amplifier and background. We define the boost factor as the ratio of the signal to noise in case (2) *versus* case (1); thus it provides a measure of the gain one can expect when employing the X-amp scheme. Fig. 5 ▸ shows representative data for this boost factor, when varying the background intensity 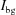 and amplifier intensity 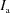, but keeping the sample scattering constant.

The contour of boost factor 1 represents the threshold where the X-amp scheme is beneficial. The limiting behavior of the boost matches what one expects intuitively from inspection of the detector images. For low backgrounds, the sample is best measured on its own, as the amplifier itself will always introduce some additional noise to the system. For higher backgrounds, stronger amplifiers are necessary in order to overcome the noise of the background. Refer to the supporting information for additional plots of the scaling of the signal-to-noise ratio for the amplifier scheme.

### 4.4. Longitudinal displacement   

The validation experiments described above located the sample and amplifier within the same plane. As a general-use technique, however, X-amp simply requires that the sample and amplifier be within the same coherence volume. As such, the amplifier can be fabricated separately, and aligned to the beam center. Samples of interest can then be conveniently translated into the beam center, whereupon they will coherently interfere with the amplifier. In the small-angle limit, the transverse coherence length (orthogonal to the beam) will be considerably smaller than the longitudinal coherence length (along the beam). For instance, the experimental setup used herein had a transverse coherence of ∼4 µm and a longitudinal coherence of ∼3 µm (Lhermitte *et al.*, 2017 ▸). Thus, while great care must be taken to co-align the sample and amplifier transversely (to bring them both within the same coherence volume), there is a large longitudinal distance over which they will coherently interfere.

## 5. Discussions   

The presented technique enables the measurement of a weakly scattering sample that is overwhelmed by strong background scattering, by introducing a designed strongly scattering amplifier. For a sufficiently strong amplifier, the interference term will itself be intense, and thus above the background noise level. This boosting thus allows otherwise unmeasurable samples to be measured. Rather than reconstruct the sample reciprocal space, we propose to exploit angular correlation analysis to recover the sample’s symmetry, which provides equivalent structural information. Crucially, this technique requires one to resolve the interference fringes arising from the coherent interference between the sample and amplifier.

For a data feature to be completely determined, it must be oversampled (Shannon, 1949 ▸). This requirement means that two points must be sampled per period of the highest-frequency term. Here, we select a measurement threshold double this baseline requirement (four points per period) to ensure we remain above this fundamental limit. In X-amp, the highest frequency components are due to the largest separation between the sample and amplifier [equation (9)]. For a reasonable setup with sample-to-detector distance of 4.81 m, pixel size 75 µm, wavelength 1.4 Å, maximum scattering wavevector 0.01 Å^−1^ (resolutions down to 62 nm) and maximum sample amplifier displacement of 2 µm, the longitudinal displacement restriction is set at 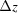 ≃ 500 µm.

A second requirement is that a high degree of coherence across the sample and amplifier must be satisfied in order to resolve the fringes. Partial coherence has the effect of blurring the fringes (Vartanyants & Robinson, 2001 ▸). For our experimental setup, we verified high coherence by measuring the diffraction of a sample 2.6 µm in spatial extent (in transverse direction), where the sample fringes were resolvable. For the longitudinal direction, coherence may be calculated as previously discussed (Lhermitte *et al.*, 2017 ▸). For the experimental setup here, longitudinal coherence is 3 mm, which is longer than the restriction set by the longitudinal displacement described above.

Another requirement is that there be no discernible coherent interference between the background source and the sample. This may be assumed true in two main cases: (1) the scattering entity generating the background is not located in the same coherence volume as the sample; (2) the background is located in the same coherence volume as the sample, but fluctuates on timescales much shorter than the sample’s. Although in this second case there is coherent interference between the background and the sample, this interference fluctuates rapidly, and may be averaged away by measuring for longer than the background fluctuation timescale.

We have so far assumed that the background obeys Poisson statistics; that is, that the variations observed in the background come from finite photon-counting statistics. In some cases, the background scattering (even if temporally static) may be non-uniform and structured. Consider, for instance, the diffuse scattering arising from windows on sample chambers (*e.g.* mica or Kapton). Such a background is temporally static but structured (even if weakly and randomly); moreover it varies randomly as the sample chamber is translated, and different parts of the window are illuminated by the coherent X-ray beam. The variance of the speckle from backgrounds collected at different positions is 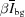 where β is the partial coherence factor (Sutton, 2008 ▸). This was observed, *e.g.* in Fig. 4 ▸, where polyimide sheets were introduced to the beam. The noise scales more unfavorably than that from Poisson statistics [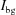 *versus* 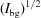]. Since the X-amp method removes the background dependence of the signal-to-noise ratio, the gains from this more unfavorable scaling are then higher.

Another limitation of the X-amp technique is the quality of the amplifier. The nanofabricated amplifier structure will contain imperfections, which will introduce spurious correlations to the analysis, both through the structuring of the amplifier scattering, and their contribution to the interference term. Thus, there are clear advantages to having extremely high-quality, defect-free amplifier structures. Moreover – obviously – there is a large advantage to fabricating amplifiers with as large a scattering power as possible. Thus, one should favor amplifiers made of strongly scattering materials (such as gold, used herein), and being as thick as possible (in the beam direction), to increase total scattering volume. Increasing the size of the amplifier in the transverse direction, however, has more limited utility. Firstly, one cannot increase the size of the object beyond the transverse coherence length. Secondly, larger amplifier objects will introduce higher-frequency components to the total scattering image, obscuring the fringing effect one desires to resolve.

Finally, the obvious limitation is the asymptotic limit referred to in equation (16), stressed throughout the paper. If the sample cannot be measured on its own without background, then it will not be visible *via* amplification. Thus, the purpose of X-amp is not to boost the sample scattering per se but rather to amplify it above a given background.

The use of an amplifier to enhance measurements of meso-clusters may or may not be of use depending on the application and details of the noise contributions. It is important to assess all the limits described here before choosing to proceed with the scheme.

## 6. Conclusion   

We have presented a technique to boost the sample scattering signal above an incoherent background. Coherent amplification cannot boost a sample’s intrinsic signal-to-noise ratio; that is, the signal-to-noise ratio for the material measured in an idealized zero background context. However, experimental X-ray scattering measurements very frequently include a non-trivial background. In the case of measuring extremely weakly scattering samples, such as finite-size meso-objects, the background scattering may overwhelm that of the sample. For instance, sample cell windows and ambient liquid – frequently present during *in-situ* measurements – unavoidably introduce background. The methodology presented here allows one to amplify sample scattering above background, and thereby allows the reconstruction of sample symmetry in cases where the sample is otherwise buried below the background scattering noise floor. We have demonstrated here on static samples how this technique can be used to amplify the signal-to-noise ratio for a sample. The presented technique is well matched to studying meso-objects in solution environments. In such a case, sample concentrations are often dilute, while the liquid environment generates a significant background; in other words, the sample scattering is overwhelmed by background. For large meso-clusters, the sample dynamics (translation and rotation in solution) will be orders of magnitude slower than the fluctuation of the liquid itself (which occurs over molecular timescales). In such a case, an exposure time can be selected that yields a non-coherent background (fluctuations averaged out) but an essentially static sample. Conceptually, X-amp can be used in such a case to repeatedly measure the sample (as it tumbles in solution), and the average of the second-order correlation curves can be used to reconstruct the sample symmetry. We also note that nano-structures and meso-structures assembled using soft linkers are typically radiation sensitive. Thus, the ability of X-amp to reduce the requisite exposure time is extremely beneficial.

The experiments presented here validate that this technique can be used to amplify sample scattering to overcome a background, and reconstruct sample symmetry. We note that the sample-to-amplifier displacement should be kept as small as possible, but that it is experimentally feasible to bring both within a coherence volume, and to minimize Ewald curvature effects to a point where robust data can be recovered. Overall, this technique may be of great utility as nanoscience delves into small, finite-size meso-objects that scatter very weakly compared to their assembly environments.

## Appendix A First-order angular correlation

This derivation demonstrates why the first-order angular correlation yields no structural information, in the limit of high background (small *X*). First, it is assumed that the sample–amplifier distance  is random and uncorrelated with the sample orientation The correlation of the terms in equation (12) is:

where  refers to the rotation matrix used to rotate the diffraction pattern by the  angle and ‘c.c.’ refers to the complex conjugate of the preceding term. Cross terms between the interference term and the amplifier or sample intensity are zero since they’re uncorrelated and the interference term averages to zero from the random phase  or . The remaining terms are:

Correlation in the amplified interference term may thus not be extracted through the use of first-order correlation functions.

## Appendix B Second-order correlation functions

This derivation demonstrates that the second-order correlation function yields the desired signal. The second-order correlation function is defined as:

Now, we can throw away a few terms. Odd moments of  will vanish due to the averaging of the random phase term  or . Next, to elucidate the desired term we make the assumptions that  and that  and  are constant along ϕ, we get just one surviving term:

The second-order correlation effectively recovers the correlation of the amplified term, which was lost in the first-order correlation.
